# Identification of factors predictive of contralateral secondary hip fractures in patients after initial hip fracture - A prospective observational study

**DOI:** 10.1016/j.bonr.2026.101908

**Published:** 2026-02-19

**Authors:** Shu Takata, Yusuke Uehara, Masaru Uragami, Tatsuki Karasugi, Tetsuro Masuda, Takayuki Nakamura, Takuya Tokunaga, Masaki Yugami, Kazuki Sugimoto, Hironori Tanoue, Satoshi Hisanaga, Ryuji Yonemitsu, Kosei Takata, Naoto Yoshimura, Yuto Shibata, Shuntaro Tanimura, Hideto Matsunaga, Makoto Tateyama, Masaki Shimada, Yuki Kai, Xiao Tian, Hikaru Goshogawa, Mizuho Yumoto, Hiroshi Takaki, Yusuke Takashima, Soichiro Karata, Takaya Funada, Yuki Uchida, Keigo Matoba, Junnosuke Ide, Ryuta Kubo, Rui Tajiri, Hibiki Yamada, Minoru Takebayashi, Naoto Kajitani, Kumamoto Study for Osteoporotic-hip fracture prevention (K-STOP) group, Takeshi Miyamoto

**Affiliations:** a Department of Orthopedic Surgery, Kumamoto University, 1-1-1 Honjo, Chuo-ku, Kumamoto, 860-8556, Japan; b Department of Oral and Maxillofacial Surgery, Kumamoto University, 1-1-1 Honjo, Chuo-ku, Kumamoto, 860-8556, Japan; c Department of Neuropsychiatry, Faculty of Life Sciences, Kumamoto University, Kumamoto, Japan

**Keywords:** Secondary hip fracture, Longitudinal study, Living alone, Three or more existing vertebral fractures

## Abstract

Hip fractures are the most serious osteoporotic fractures, and patients often develop contralateral hip fractures. Various factors are reported as risks for secondary hip fracture, but risk analysis is not yet standardized. We conducted a longitudinal prospective observational study of 1395 hip fracture patients followed for 345.2 ± 189.3 (3–795) days to identify risk factors for secondary hip fracture. Of the initial 1395 patients, we followed 1223 cases, excluding those who already had a contralateral hip fracture at time of enrollment. Univariate analysis using the log-rank test and Cox regression analysis of 51 factors such as age, BMI and living alone in relation to secondary hip fracture showed that four factors, namely, living alone, low grip strength (<18 kg), three or more existing vertebral fractures and hypertension, were significant risks for secondary fracture. Multivariate Cox regression analysis using factors identified as significant risks for secondary fracture in univariate analysis confirmed that living alone and the presence of three or more existing vertebral fractures could be useful in predicting risk for secondary hip fracture. Our results may provide important knowledge to prevent contralateral secondary hip fractures in patients after the first fracture.

## Highlights

• Primary hip fracture cases were followed to identify risks for secondary hip fracture.
• Survival analysis revealed two independent risk factors for secondary hip fracture.
• Case comparison revealed three independent risk factors for secondary hip fracture.
• Living alone was identified as a risk for secondary hip fracture by both analyses.
• Risk factors differ between primary and contralateral secondary hip fractures.

## 1 Introduction

Hip fracture is the most serious osteoporotic fragility fracture occurring in the elderly (Nazrun et al., 2014). Hip fracture causes reduced activity of daily living (ADL) and decreased quality of life (QOL), and affected patients require care even after surgical treatment (Peeters et al., 2016; Mariconda et al., 2016). Also, after the primary hip fracture, secondary hip fractures of the contralateral side not only promote a further decline in ADL and QOL, but can put a patient at risk of more serious conditions or even death (Sobolev et al., 2015). Moreover, secondary hip fractures are problematic from a health economics perspective (Williamson et al., 2017).

Secondary hip fractures occurring in the year after first fracture are seen in 3.4–3.8% of primary hip fracture patients in Japan (Hagino et al., 2012; Yamanashi et al., 2005). Other recent reports based on databases available from other countries report comparable recurrence rates of 1.24% or 2.5% (Ho and Wong, 2020; Kay et al., 2024). The time elapsed from the first hip fracture to the secondary hip fracture is reportedly within a year or even several months of the first hip fracture (Nymark et al., 2006), indicating that secondary fractures often occur early. In fact, in our previous study enrolling 1395 patients with hip fracture, excluding high-energy trauma or fractures due to tumors, 172 patients (12.3%) already exhibited contralateral hip fracture at enrollment (Uragami et al., 2023).

Various risk factors for hip fracture are known and include low bone mineral density and factors identified using the Fracture Risk Assessment Tool (FRAX; The University of Sheffield, Sheffield, UK) algorithm, such as rheumatoid arthritis (Kanis et al., 2007). However, until recently, no known studies have evaluated these factors in a comprehensive manner. However, we recently identified ten factors significantly associated with hip fracture, namely, low vitamin D status, low bone mineral density at the femoral neck, low Barthel index score, low hand grip strength, locomotive syndrome, increased number of previous falls, low serum IGF1 levels, consumption less than five cups of tea per day, non-use of anti-osteoporotic agents and low body mass index (BMI) (Uragami et al., 2023). Due to these efforts, it is now possible to screen and identify individuals at risk for developing hip fracture prior to its onset.

Based on information from the American Society for Bone and Mineral Research/Bone Health & Osteoporosis Foundation (ASBMR/BHOF) task force, interventions with anti-osteoporotic drugs such as an osteoanabolic agent are now recommended as goal-directed osteoporosis treatment for patients with hip fracture to prevent secondary fractures (Cosman et al., 2024). However, few studies have evaluated various risks of secondary hip fracture in a comprehensive and prospective manner.

Factors such as female gender (Ryg et al., 2009), advanced age (Zhu et al., 2014) and low body mass index (Stewart et al., 1999) are reported risks of secondary hip fracture, as are other factors, such as: fracture type (Kim et al., 2012), living in a nursing home (Zhu et al., 2014), living alone (Ryg et al., 2009), walking with an aid (Larrainzar-Garijo et al., 2022), history of falls (Larrainzar-Garijo et al., 2022), less walking time (Chapurlat et al., 2003), previous fragility fracture (Ryg et al., 2009), cognitive impairment (Zidrou et al., 2023), excessive alcohol intake (Ryg et al., 2009), low femoral neck T-score (Stewart et al., 1999), and non-use of anti-osteoporosis drugs (Morin et al., 2007). Several comorbidities including cerebrovascular disease (Shen et al., 2014), Parkinson's disease (Zhu et al., 2014), visual impairment (Zhu et al., 2014), cardiovascular disease (Zhu et al., 2014), respiratory disease (Zhu et al., 2014), chronic liver disease (Mazzucchelli et al., 2018), hypertension (Shen et al., 2014), dyslipidemia (Shen et al., 2014), and diabetes (Shen et al., 2014) are also reported to be risks for secondary hip fracture.

Here, to clearly define factors predictive of secondary hip fracture, we performed a longitudinal prospective observational study of 1395 patients with hip fracture and followed 1223 of them for 345.2 ± 189.3 (3–795) days, excluding patients who already exhibited contralateral hip fracture at the time of enrollment. We then assessed potential risk factors in patients who had contralateral hip fracture during the observation period. During that time, 37 patients developed contralateral secondary fractures. Based on that analysis we identified and report three risk factors for secondary hip fracture.

## 2 Materials and methods

### 2.1 Study design and participants

The Kumamoto STudy for Osteoporotic-hip fracture Prevention (K-STOP) group was organized to conduct studies aimed at preventing new hip fractures. K-STOP includes 23 hospitals associated with the Department of Orthopedic Surgery of Kumamoto University, and hip fracture cases were recruited from patients admitted to 21 of those hospitals. Each facility registered participants during a one-year period falling between March 2021 and November 2022. Excluded were fracture patients who: (1) were under age 20, (2) refused to participate, (3) had a pathological fracture, or (4) had a fracture due to industrial or traffic accidents. In total, 1395 hip fracture patients were enrolled in the group.

This study was initially approved by the Kumamoto University Institutional Review Board and then by the ethics committee of each research institute. This study is a prospective observational study of 1395 hip fracture patients enrolled in the K-STOP study.

### 2.2 Data collection

Data was collected using a standardized two-part questionnaire and measurement methods across all 21 research sites. All participants responded to questions and were examined while hospitalized.

The first part of the questionnaire included self-reported responses to questions administered by medical stuff at each facility. Proxies such as family members or other caregivers were allowed to respond only if subjects showed cognitive impairment or other health problems, such as visual impairment. Review of medical records determined whether questionnaires contained insufficient data. The second part of the questionnaire was administered by medical staff at each site using the subject's medical record and examinations. Those questions pertained to body mass index (BMI); current use of anti-osteoporosis drugs (bisphosphonates, selective estrogen receptor modulators, parathyroid hormone peptides, alfacalcidol, eldecalcitol, denosumab, romosozumab, or other drugs); use of glucocorticoids or proton pump inhibitors; maximum handgrip strength, previous hip fractures, previous vertebral fractures, femoral neck T-score, and serum IGF-1 and 25(OH)D levels. The serum IGF-1 and 25(OH)D levels were measured by electro chemiluminescence immunoassay and chemiluminescent enzyme immunoassay, respectively. Height and weight were measured and BMI calculated as weight divided by height squared (kg/m2). Handgrip strength was measured twice for each hand using a strength dynamometer according to instructions provided by trained personnel or a nurse. After participants exerted maximal handgrip strength, and the maximum value between the two hands was used, and results were expressed in kilograms. Participants experiencing pain in hands and/or elbows or with severe cognitive impairment prohibiting them from following instructions were excluded from handgrip assessment. The dynamometers adopted by each facility were used in this study to evaluate handgrip strength. Previous hip fractures were defined as detection of a contralateral hip fracture in the case group based on plain radiographs of bilateral hips in an anteroposterior view. Radiographs of bilateral hips were also reviewed in the control groups. Previous vertebral fractures were confirmed by lateral views of the whole spine and assessed semi-quantitatively, such that a vertebral body was considered fractured if it was graded 1 or higher (Genant et al., 1993). Fragility fractures were defined as fractures satisfying any of the following criteria: radiographic examination revealing previous vertebral fractures, previous hip fractures, or self-reporting of fragility fractures in the first questionnaire (i.e., proximal humerus or distal radius fractures occurring at other sites). Bone mineral density was measured at the femoral neck by dual-energy X-ray absorptiometry.

Finally, we examined a registry of patients who survived one year after their first hip fracture to determine which patients remained alive. We then sent surviving patients letters from the research office of Kumamoto University Hospital inquiring about the presence and type of new fractures, their date of occurrence, and current osteoporosis treatment and type. Participants who did not return the survey were telephoned and asked to complete the questionnaire.

### 2.3 Statistical analysis

This analysis compared 51 factors collected at enrollment in the K-STOP study between the group that experienced a secondary fracture within one year and the group that did not. Additionally, the initial hip fracture admission date was designated as the observation start date, with the primary outcome defined as the occurrence of a secondary hip fracture. The observation endpoint was either the event occurrence date or the observation completion date. The study was terminated if no events were observed by the end of the observation period. The follow-up period for patients after their initial fracture ranged from 3 to 795 days. Deaths were treated as censored events and were not considered competing risks. Initially, we performed univariate analyses for each variable (see Supplementary Table 1) using Cox regression analysis. For binary variables, survival curves were generated using the Kaplan–Meier method and compared with the log-rank test. A *p*-value < 0.05 was considered statistically significant. Using variables identified as significant risk factors in univariate analysis, we then performed multivariate Cox regression analysis, in which the proportional hazards assumption was evaluated using scaled Schoenfeld residuals for each covariate. In univariate Cox proportional hazards regression analysis, *p*-values for factors that reached statistical significance were adjusted using the Benjamini–Hochberg method to control the false discovery rate (FDR) correction, and the corresponding q-values were calculated. A q-value < 0.05 was considered statistically significant.

Also, among patients who survived at 365 days post-initial fracture and exhibited clear status developed a contralateral fracture within 365 days, while the other group (the control group) did not. Univariate group comparisons were made between case and control groups using Fisher's exact test for categorical variables and Welch's *t*-test for continuous variables. Logistic regression analysis adjusted for age, gender, and BMI was also performed. A *p*-value <0.05 was considered significant. Multivariate logistic regression analysis was performed with significant factors as explanatory variables. Data collection and analysis were performed using R version 4.2.1 (www.r-project.org).

## 3 Results

### 3.1 Participants

**Fig. 1:**
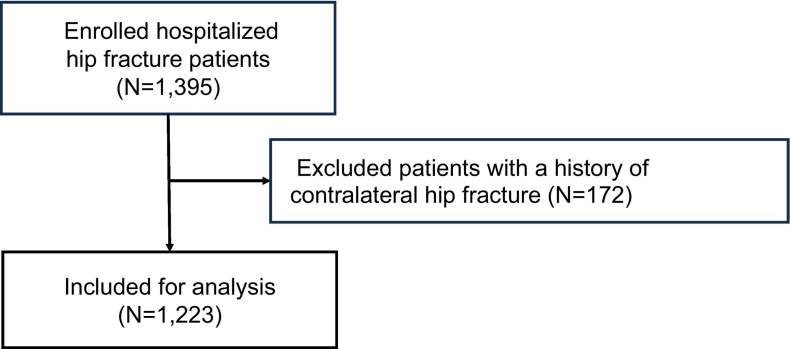
Patient flowchart**.** We enrolled 1395 hip fracture patients. Among them, 172 with a history of contralateral fractures were excluded. The remaining 1223 patients were followed for 345.2 ± 183.9 days.

**Fig. 2:**
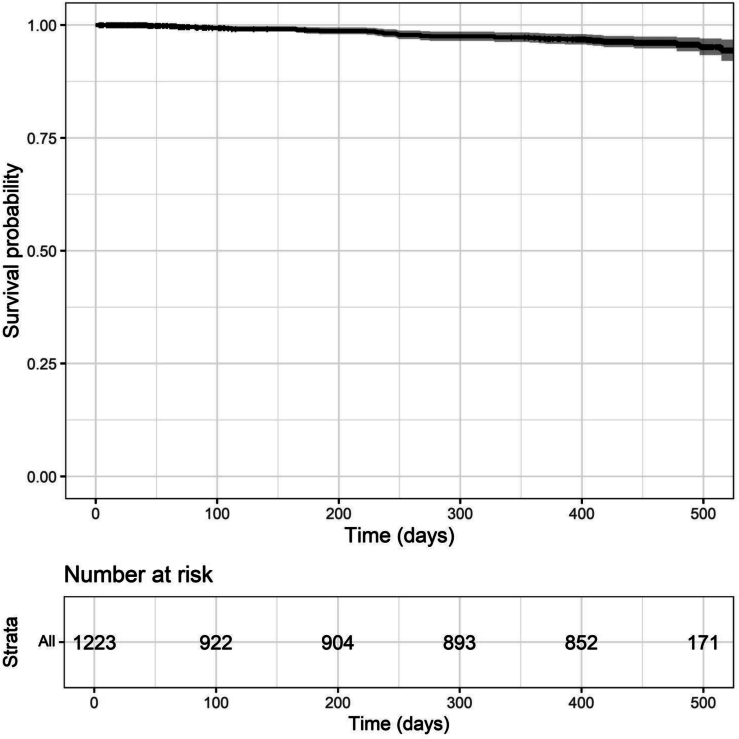
Kaplan-Meier plots of incidence of secondary hip fracture after initial hip fracture. Kaplan-Meier plots showing the time interval from the initial hip fracture to the secondary hip fracture.

The goal of this study was to assess hip fracture patients for factors that put them at risk of secondary hip fracture. To do so, first we evaluated data from 1395 fragility hip fracture patients previously enrolled in the K-STOP study. Among them, 172 who already exhibited contralateral side hip fracture at the time of enrollment were excluded. Thus, we followed the remaining 1223 subjects (Fig. 1), for a mean observation period of 345.2 days (standard deviation 189.3). Of those, 37 developed contralateral secondary fractures during that period (Fig. 2).

### 3.2 Identification of risk factors predictive of secondary hip fracture by univariate Cox regression analysis

**Fig. 3:**
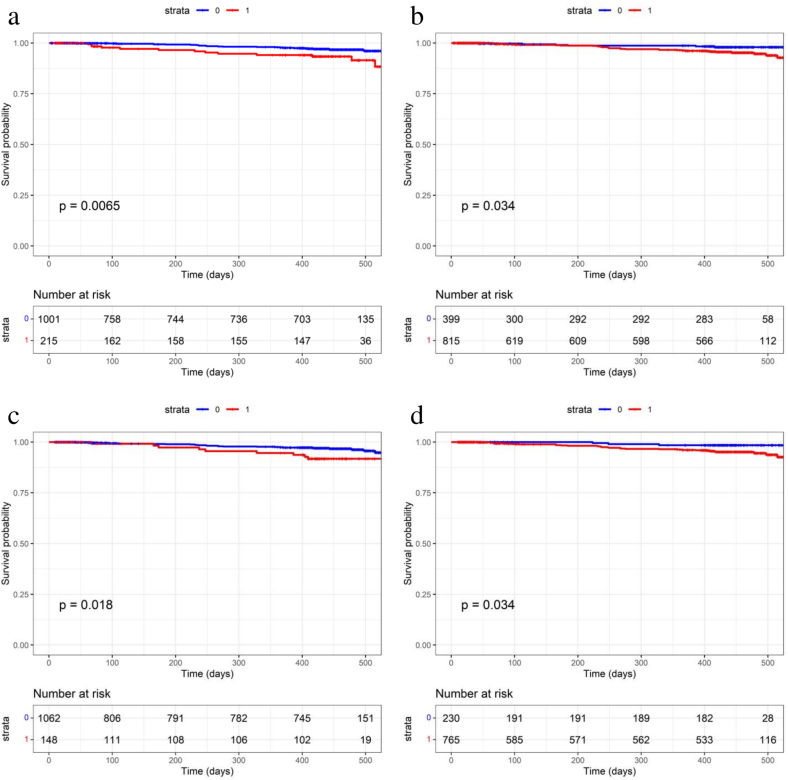
Kaplan-Meier plots of the incidence of secondary hip fracture in patients with indicated risk factor. Kaplan-Meier plots showing the incidence of secondary hip fracture in the absence or presence of a) living alone, b) a maximal handgrip strength <18 kg, c) existing vertebral fractures in 3 or more vertebral bodies, and d) hypertension. *0*: absence, *1*: presence.

Next, we performed univariate analyses for each variable (see Supplementary Table 1) using Cox regression analysis in 1223 subjects to extract risk factors predictive of secondary hip fracture (Table 1). That analysis showed that living alone (HR 2.48, 95% CI 1.26–4.87, *p* = 0.0085), low maximal grip strength (<18 kg) (HR 3.34, 95% CI 1.02–10.93, *p* = 0.046), three or more existing vertebral fractures (HR 2.41, 95% CI 1.14–5.10, *p* = 0.022) and hypertension (HR 2.49, 95% CI 1.04–5.97, *p* = 0.041) were significant risks for contralateral secondary hip fracture (Table 1). Fig. 3 shows Kaplan-Meier curves for each factor identified by these analyses.

**Table 1:** Univariate Cox regression analysis.

Variables	HR	95%CI	P-value
Living alone	2.48	1.261–4.867	0.0085
Maximal handgrip strength <18 kg	3.34	1.021–10.930	0.0462
3 or more existing vertebral compression fractures	2.41	1.135–5.099	0.0220
Hypertension	2.49	1.039–5.969	0.0408

Only significant factors are listed.

### 3.3 Multivariate Cox regression analysis of factors identified as risks for secondary hip fracture in univariate analyses

**Fig. 4:**
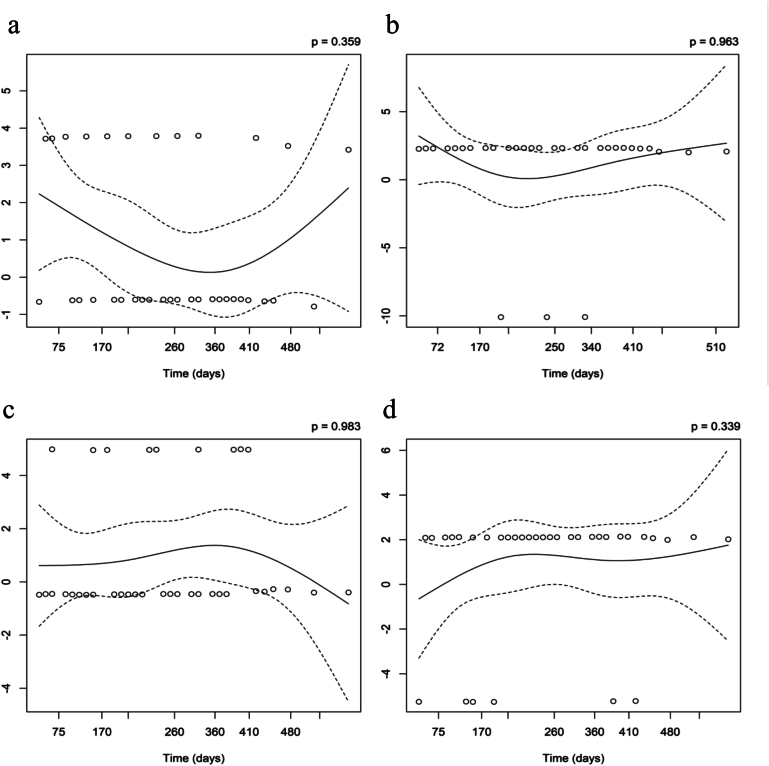
Proportional Hazards for secondary hip fracture in patients with an indicated risk factor. Analysis of Proportional Hazards Based on Schoenfeld Residuals of patients a) living alone, b) showing a maximal handgrip strength <18 kg, c) having existing vertebral fractures in 3 or more vertebral bodies, and d) hypertension. The vertical axis shows the time-varying coefficient β(t) corresponding to each factor.

Finally, we performed multivariate Cox regression analysis using factors identified as risks for secondary hip fracture in univariate analyses. After applying false discovery rate (FDR) correction using the Benjamini–Hochberg method, all factors identified as significant risks in the univariable Cox proportional hazards regression analysis remained statistically significant (Supplementary Table 2). Multivariate Cox regression analysis confirmed that two of the four factors identified as risk factors by univariate analyses, namely, living alone (HR 2.16, 95% CI 1.07–4.38, *p* = 0.032) and three or more existing vertebral fractures (HR 2.37, 95% CI 1.10–5.11, *p* = 0.028), were independent risks for secondary hip fracture (Table 2). The proportional hazards assumption was tested using Schoenfeld residuals. No significant violation was detected for any covariate (global test: chi-squared = 0.513, degrees of freedom = 4, *p*-value = 0.97), indicating that the proportional hazards assumption was reasonably met (Fig. 4 and Table 3).

**Table 2:** Multivariate Cox regression analysis of factors significant in univariate Cox regression analysis.

Variables	HR	95%CI	P-value
Living alone	2.16	1.067–4.378	0.0324
Maximal handgrip strength <18 kg	3.01	0.913–9.940	0.0702
3 or more existing vertebral compression fractures	2.37	1.100–5.113	0.0276
Hypertension	2.47	0.953–6.383	0.0629

**Table 3:** Test of proportional hazards assumption using Schoenfeld residuals.

Covariate	chi-squared	degrees of freedom	P-value
Living alone	0.223	1	0.64
Maximal handgrip strength <18 kg	3.84e-4	1	0.98
3 or more existing vertebral compression fractures	9.59e-5	1	0.99
Hypertension	0.253	1	0.61
Global	0.513	4	0.97

## 4 Discussion

Contralateral secondary hip fracture promotes not only a significant loss of ADL and QOL, but is problematic in terms of medical economics. Thus, it is mandatory to prevent these injuries when possible. In an effort to better predict these events, we re-analyzed patient data initially acquired in a previous study of primary fractures (Uragami et al., 2023) and followed patients who had an initial hip fracture as a longitudinal cohort study. That analysis identified two independent factors, namely, living alone and three or more existing vertebral fractures by Multivariate Cox regression analyses as risk factors for contralateral secondary hip fracture.

Living alone is reportedly associated with hip fracture risk, with a one-year mortality rate after hip fracture (MacRae et al., 2024). Hip fracture is associated with decreased ADL (Yeritsyan et al., 2024), and the combination of living alone with decreased ADL after a primary fracture may increase a patient's risk of falling and subsequent re-fracture. This finding strongly suggests that patients require assistance after hip fracture, even after surgery. In fact, living alone reportedly increases the risk of admission to assisted-living facilities (MacRae et al., 2024). On the other hand, a prospective study found that the risk of a secondary hip fracture increases with higher functional status (Berry et al., 2007). Elderly individuals living alone may have higher functional status compared to those in other living environments, and this could be associated with a higher risk of falling. However, several contexts such as caregiver support are not incorporated in this study.

The presence of fracture dominoes indicates that small fractures, such as those occurring in the distal radius, can gradually lead to more serious ones in the case of vertebral or hip fractures (Klotzbuecher et al., 2000). These observations underscore the need to prevent a subsequent fragility fracture while the first fracture remains minor. On the other hand, unlike a fracture domino, multiple fractures reportedly can be exhibited at a single site, as seen in a patient who, having experienced an initial distal radius fracture, repeatedly injured the same site (Izquierdo-Avino et al., 2023). Here, we identified three or more existing vertebral fractures as a risk factor for contralateral secondary hip fractures. The presence of three or more vertebral fractures may reflect a high degree of frailty. Indeed, it has been reported that having one vertebral fracture is associated with a higher risk of subsequent fragility fractures than having none, and that having two vertebral fractures confers a higher risk than having one (Lindsay et al., 2001). Among patients with an initial hip fracture, those with three or more vertebral fractures are therefore likely to exhibit more severe bone fragility and were identified as being at higher risk of contralateral secondary fractures.

Advanced age, greater BMI, and prevalence of comorbidities have been identified as risk factors for patients presenting with periprosthetic femoral fractures (PFF), and PFF has been reported as an osteoporotic fracture (Houel et al., 2025). Among the risk factors for contralateral secondary fractures identified in our Cox regression analyses for hip fractures—namely, living alone and the presence of three or more pre-existing vertebral fractures—the latter shares the common feature of reflecting bone fragility. Furthermore, in the two-group comparison between fracture and non-fracture groups in our study, the fracture group was significantly older than the non-fracture group. This point also indicates a commonality between the risk factors for PFF and those for contralateral secondary fractures. In contrast, higher BMI and prevalence of comorbidities, which have been identified as risk factors for PFF, were not significantly associated with contralateral secondary fractures in our study. These findings suggest that while some risk factors for PFF and contralateral secondary fractures after hip fracture overlap, there are also differences between them.

In this longitudinal cohort study, to assess risk of developing a contralateral hip fracture, we identified risk factors for contralateral secondary fractures using survival analysis, which included individuals who died or relocated within one year of the initial fracture. However, we also considered it important to identify risk factors for contralateral secondary fractures in individuals likely to survive at least one year after an initial hip fracture and to suggest strategies for their prevention. To this end, among the 1395 hip fracture cases enrolled in the K-STOP study, we excluded: 172 with a contralateral hip fracture at enrollment, 162 who died within one year of fracture, and 142 who could not be followed due to relocation or missing data. After evaluating the remaining 919 participants for occurrence of a contralateral secondary fracture within a year of initial fracture, we identified 31 who suffered a secondary fracture and 888 who did not (Supplementary Fig. 1). We then adjusted factors listed in Supplementary Table 1 for age, gender, and BMI, and performed logistic regression analysis to extract risk factors for secondary hip fracture (Supplementary Table 3) and found that having a femoral neck fracture at the first fracture was a significant risk for a secondary hip fracture (OR 3.29, 95% CI 1.460–7.430, *p* = 0.0042), as was living alone (OR 3.60, 95% CI 1.710–7.580, *p* = 0.0007) (Supplementary Table 4). A lower eGFR level was also confirmed a significant risk for secondary hip fracture (OR 0.98, 95% CI 0.965–0.999, *p* = 0.0336) (Supplementary Table 4).

We then performed multivariate logistic regression analysis using factors identified as risks for secondary hip fracture in a two-group comparison or in logistic regression analysis. Significant differences in age and sex, as well as low serum 25(OH)D and IGF1 levels—all identified by comparison of the two groups—were not confirmed by multivariate logistic regression analysis (Supplementary Table 5), which instead identified femoral neck fracture as a fracture type at the first fracture (OR 4.36, 95% CI 1.830–10.400, *p* = 8.65 × 10^−4^), living alone (OR 3.99, 95% CI 1.820–8.760, *p* = 5.43 × 10^−4^) and low eGFR status (OR 0.976, 95% CI 0.957–0.995, *p* = 0.012) as independent risks for secondary hip fracture (Supplementary Table 5).

Hip fractures are generally of two types: femoral neck fracture and femoral trochanteric fracture. The latter occurs more frequently than femoral neck fractures in older patients (Hagino et al., 2017) and is reportedly a risk factor for contralateral secondary hip fractures (Hagino et al., 2012). It is reasonable to assume that femoral trochanteric fracture occurring in older patients would be a risk for contralateral secondary hip fracture, given that fracture risk reportedly increases with age (Takusari et al., 2021). However, here we found that femoral neck rather than trochanteric fracture was an independent risk factor for contralateral secondary hip fracture, even after adjusting for age or applying multivariate logistic regression analysis, and the OR was 4.36. Femoral neck fractures reportedly predominate at the lowest structural mechanical strength levels, whereas trochanteric fractures are more common at high failure loads (Pulkkinen et al., 2006). These observations support the idea that femoral neck fracture is a likely risk for contralateral secondary hip fracture.

Although low eGFR has been reported to be a risk for hip fracture (Goto et al., 2020), it has not been considered a risk for contralateral secondary fracture after an initial hip fracture. In this study, the cut-off eGFR value for development of contralateral secondary fracture of the hip was found to be 59 ml/min/1.73m^2^ (Supplementary Fig. 2), a value corresponding to stage G3a of Chronic Kidney Disease (CKD). CKD due to reduced renal function is a known risk factor for CKD-Mineral and Bone Disorder (CKD-MBD) (Fried et al., 2007), and low eGFR may increase bone fragility and risk for contralateral secondary fracture. Our results also show that eGFR levels are associated with risk of contralateral secondary hip fracture, and the lower the eGFR levels, the greater that risk (data not shown).

Although many factors have been reported as risk factors for hip fracture, in our study, none of the 10 factors previously characterized as most strongly associated with development of hip fracture—namely, serum 25(OH)D levels, femoral neck T-score, the Barthel index score, maximal handgrip strength, GLFS-25 score, number of falls in the previous 12 months, serum IGF-1 levels, number of cups of tea/day, use of anti-osteoporosis drugs, and BMI (Uragami et al., 2023)—were identified as risk factors for contralateral secondary hip fractures. Although osteoporosis treatment is thought to reduce hip fracture risk (Murad et al., 2012), neither the rate of osteoporosis patients treated with anti-osteoporotic agents before the first fracture nor the rate of those at discharge after fracture differed significantly between patients with or without contralateral secondary hip fracture. These results suggest that risk factors for contralateral secondary hip fracture differ from those for initial hip fracture.

We demonstrated that Cox regression analyses and logistic regression analysis both identified living alone as a common risk factor for contralateral secondary fractures, whereas other risk factors differed between the two analytical approaches. Although the presence of three or more pre-existing vertebral fractures was identified as a risk factor for contralateral secondary fractures in the Cox regression analyses, the two-group comparison showed a higher prevalence in the fracture group (22.6%) than in the non-fracture group (11.9%); however, this difference did not reach statistical significance (*p* = 0.090). Thus, while the exact reasons for the discrepant results between the analytical methods remain unclear, the logistic regression analysis—which included only individuals who survived at least one year after the initial fracture—had a smaller sample size than the Cox regression analyses (Cox regression analyses, *N* = 1223; logistic regression analysis, *N* = 919). Factors influencing individuals who died within one year after the initial fracture may therefore have affected the results.

A limitation of this study is the small number (37) of contralateral secondary hip fracture cases, indicating a need to enroll and follow up additional primary hip fracture cases to better assess risk of developing a contralateral secondary hip fracture. The occurrence of contralateral secondary hip fracture was captured through questionnaires or phone calls in this study, and thus, under-reporting in the occurrence of contralateral secondary hip fractures may bias the results. Although there are reports of large-scale studies using data acquired nationwide as a means to significantly increase case number, it is difficult to conduct studies specific to the risk of hip fracture and challenging to comprehensively assess all risk factors for hip fracture.

It was unclear whether any patients who died within one year after the initial fracture had a secondary fracture. However, we considered it critical to assess potential risk factors for contralateral secondary hip fractures to prevent further fractures in cases surviving at least one year after the initial fracture.

Taken together, our study suggests that careful follow-up, such as through patient liaison services and pharmaceutical and care interventions may be able to prevent contralateral secondary hip fractures in patients by monitoring factors identified here.

## CRediT authorship contribution statement

**Shu Takata:** Writing – original draft, Visualization, Project administration, Investigation, Funding acquisition, Formal analysis, Data curation. **Yusuke Uehara:** Supervision, Project administration. **Masaru Uragami:** Resources, Project administration, Methodology, Data curation. **Tatsuki Karasugi:** Supervision, Project administration. **Tetsuro Masuda:** Supervision, Project administration. **Takayuki Nakamura:** Supervision, Project administration. **Takuya Tokunaga:** Supervision, Project administration. **Masaki Yugami:** Supervision. **Kazuki Sugimoto:** Supervision. **Hironori Tanoue:** Supervision. **Satoshi Hisanaga:** Supervision. **Ryuji Yonemitsu:** Supervision. **Kosei Takata:** Supervision. **Naoto Yoshimura:** Supervision. **Yuto Shibata:** Data curation. **Shuntaro Tanimura:** Supervision. **Hideto Matsunaga:** Supervision. **Makoto Tateyama:** Supervision. **Masaki Shimada:** Supervision. **Yuki Kai:** Supervision, Investigation. **Xiao Tian:** Supervision. **Hikaru Goshogawa:** Supervision, Investigation. **Mizuho Yumoto:** Investigation. **Hiroshi Takaki:** Data curation. **Yusuke Takashima:** Data curation. **Soichiro Karata:** Data curation. **Takaya Funada:** Supervision. **Yuki Uchida:** Data curation. **Keigo Matoba:** Data curation. **Junnosuke Ide:** Data curation. **Ryuta Kubo:** Supervision. **Rui Tajiri:** Supervision. **Hibiki Yamada:** Supervision. **Minoru Takebayashi:** Project administration, Conceptualization. **Naoto Kajitani:** Project administration, Conceptualization. **Takeshi Miyamoto:** Writing – review & editing, Supervision, Project administration, Methodology, Funding acquisition.

## Declaration of competing interest

The authors declare that they have no known competing financial interests or personal relationships that could have appeared to influence the work reported in this paper.

## Data Availability

The authors do not have permission to share data.

## Appendix A

Members of the K-STOP group (in alphabetical order based on affiliation) include: Dr. Koichiro Oe (Amakusa Medical Center), Dr. Yuichiro Oda (Arao Municipal Ariake Medical Center), Dr. Toshitake Yakushiji (Hitoyoshi Medical Center), Dr. Mitsunobu Watanabe (Konan Hospital), Dr. Yasuyuki Abe (Kumamoto Chuo Hospital), Dr. Takaaki Sagara (Kumamoto City Hospital), Dr. Kenshi Kikukawa (Kumamoto General Hospital), Dr. Hiromichi Yasuoka (Kumamoto Kenhoku Hospital), Dr. Hidechika Nakashima (Kumamoto Kinoh Hospital), Dr. Tetsuya Fukumoto (Kumamoto Medical Center), Dr. Takuya Ikuta (Kumamoto Orthopaedic Hospital), Dr. Kazuhiko Miyamoto (Kumamoto Red Cross Hospital), Dr. Takashi Ikeda (Kumamoto Rosai Hospital), Dr. Hiroomi Ogata (Kumamoto Saishun Medical Center), Dr. Makoto Miyazaki (Minamata City Hospital and Medical Center), Dr. Hidehiko Matsushita (Omuta Tenryo Hospital), Dr. Yoshihisa Anraku (Saiseikai Kumamoto Hospital), Dr. Hiroshi Tsurukami (Tsurukami Clinic of Orthopedics and Rheumatology), Dr. Satoshi Ootawa (Uki General Hospital), and Dr. Satoshi Kudo (Yamaga Medical Center).

## Appendix B Supplementary data

Supplementary tables Image 1

Fig. S1 Patient flowchart. We enrolled 1395 hip fracture patients and followed them for 1 year. Excluded were those with a history of contralateral hip fracture, those who died within 1 year of the initial fracture, and those who could not be followed for various other indicated reasons. Of the remaining 919 patients, 31 sustained contralateral hip fractures in the follow-up year, while 888 did not. Fig. S2 Cut-off value for eGFR levels significantly associated with occurrence of secondary hip fracture within 1 year. Cut-off values for eGFR levels significantly associated with occurrence of secondary hip fracture within 1 year were 59 ml/min/1.73m^2^, based on the Receiver Operating Characteristic (ROC) curve. Area Under Curve (AUC) was determined to be 0.628 (95% CI, 0.539–0.716). Image 2
