# Supplementary material for: Identification of factors predictive of contralateral secondary hip fractures in patients after initial hip fracture - A prospective observational study
Source: Bone Rep. 2026 Feb 19;29:101908. doi: 10.1016/j.bonr.2026.101908 (PMC12973514; doi:10.1016/j.bonr.2026.101908)
Supplement: Supplementary file 1 — Supplementary tables [file mmc1.docx]

**Supplementary tables**

Supplementary table 1. Explanatory variables used in the analysis

Previously identified risk factors for secondary hip fractures

・Sex (binary variable; male/female) [1]

・Age at study entry (continuous) [2]

・BMI (kg/m²) (continuous) [3]

・Fracture pattern (binary variable; femoral neck fracture/femoral intertrochanteric fracture) [4]

・Living in a nursing home (binary variable; yes/no) [2]

・Living alone (binary variable; yes/no) [1]

・Walking with an aid (binary variable; yes/no) [5]

・Number of falls in previous 12 months (none, 1, 2, 3 or more) [5]

・Hours per week spent walking (inability to walk,<1, 1-4, ≥4) [6]

・Previous fragility fractures (binary variable; yes/no) [1]

・Cognitive impairment (no significant, mild cognitive impairment, somewhat difficult to communicate, difficulty in communicating, communication extremely difficult, completely unable to communicate) [7]

・Cerebrovascular disease (cerebral infarction, cerebral hemorrhage, subarachnoid hemorrhage, transient ischemic attack, other cerebrovascular disease) (binary variable; yes/no) [8]

・Parkinson's disease (binary variable; yes/no) [2]

・Visual impairment (binary variable; yes/no) [2]

・Cardiovascular disease (ischemic heart disease, heart failure, other cardiovascular disease) (binary variable; yes/no) [2]

・Respiratory diseases (pneumonia, chronic obstructive pulmonary disease, asthma, other respiratory diseases) [2]

・Chronic liver disease (binary variable; yes/no) [9]

・Hypertension (binary variable; yes/no) [8]

・Dyslipidemia (serum LDL-cholesterol≧140mg/dL, serum triglyceride≧150mg/dL, HDL-cholesterol＜40mg/dL) (binary variable; yes/no) [8]

・Diabetes (binary variable; yes/no) [8]

・Alcohol intake (3 or more units per day) (binary variable; yes/no) [1]

・Femoral neck T-score (continuous) [3]

・Use of anti-osteoporosis drugs before initial fracture (binary variable; yes/no) [10]

・Use of anti-osteoporosis drugs when discharged from hospital (binary variable; yes/no) [10]

Variables associated with higher risk of hip fractures

・History of hip fracture in a first degree relative (binary variable; yes/no) [11]

・Rheumatoid arthritis (binary variable; yes/no) [11]

・Secondary osteoporosis (binary variable; yes/no) [11]

・Glucocorticoid use (binary variable; yes/no) [11]

・Smoking status (non-smoker, current smoker, smoking cessation < 10 years, smoking cessation ≥ 10 years) [11]

・Number of existing compression fractures (one or more, two or more, three or more) (binary variable; yes/no) [11]

・Cups of tea/day (none, one, two, three, four, more than five) [12]

・Hyperthyroidism (binary variable; yes/no) [13]

・Chronic liver disease (binary variable; yes/no) [14]

・Chronic kidney disease (binary variable; yes/no) [15]

・eGFR (ml/min/1.73 ㎡) (continuous) [15]

・Gastric surgery (binary variable; yes/no) [16]

・Proton pump inhibitor use (binary variable; yes/no) [17]

・Serum 25(OH)D (ng/ml) (continuous) [18]

・Serum IGF-1 (ng/ml) (continuous) [19]

Variables associated with higher risk of falls

・Has Long-term Care Insurance (LTCI) certification (binary variable; yes/no)[20]

・Nocturia (no waking up during the night to urinate, waking up once or twice, waking up

three or four times, waking up more than five times, use of a diaper) [21]

・Use of hypnotics (binary variable; yes/no) [22]

・Lumbar spinal canal stenosis (binary variable; yes/no) [23]

・Type of bed (binary variable; western bed/Japanese futon) [24]

・The 25-question Geriatric Locomotive Function Scale (GLFS-25) score (continuous) [25]

・Barthel index score (continuous) [26]

・Maximal handgrip strength (binary variable ;<18kg, 18kg or more) [27, 28]

[1] J. Ryg, L. Rejnmark, S. Overgaard, K. Brixen, P. Vestergaard, Hip fracture patients at risk of second hip fracture: a nationwide population-based cohort study of 169,145 cases during 1977-2001, J Bone Miner Res 24(7) (2009) 1299-307.

[2] Y. Zhu, W. Chen, T. Sun, Q. Zhang, J. Cheng, Y. Zhang, Meta-analysis of risk factors for the second hip fracture (SHF) in elderly patients, Arch Gerontol Geriatr 59(1) (2014) 1-6.

[3] A. Stewart, L.G. Walker, R.W. Porter, D.M. Reid, W.R. Primrose, Predicting a second hip fracture, J Clin Densitom 2(4) (1999) 363-70.

[4] S.M. Kim, Y.W. Moon, S.J. Lim, B.K. Yoon, Y.K. Min, D.Y. Lee, Y.S. Park, Prediction of survival, second fracture, and functional recovery following the first hip fracture surgery in elderly patients, Bone 50(6) (2012) 1343-50.

[5] R. Larrainzar-Garijo, A. Díez-Pérez, E. Fernández-Tormos, D. Prieto-Alhambra, Risk factors for a second nonsimultaneous hip fracture in a prospective cohort study, Arch Orthop Trauma Surg 142(10) (2022) 2611-2617.

[6] R.D. Chapurlat, D.C. Bauer, M. Nevitt, K. Stone, S.R. Cummings, Incidence and risk factors for a second hip fracture in elderly women. The Study of Osteoporotic Fractures, Osteoporos Int 14(2) (2003) 130-6.

[7] C. Zidrou, A.V. Vasiliadis, S. Rizou, A. Beletsiotis, Second hip fracture in older adults: incidence and risk factors, Eur J Orthop Surg Traumatol 33(5) (2023) 1599-1606.

[8] S.H. Shen, K.C. Huang, Y.H. Tsai, T.Y. Yang, M.S. Lee, S.W. Ueng, R.W. Hsu, Risk analysis for second hip fracture in patients after hip fracture surgery: a nationwide population-based study, J Am Med Dir Assoc 15(10) (2014) 725-31.

[9] R. Mazzucchelli, E. Pérez-Fernández, N. Crespí, A. García-Vadillo, G. Rodriguez Caravaca, A. Gil de Miguel, L. Carmona, Second Hip Fracture: Incidence, Trends, and Predictors, Calcif Tissue Int 102(6) (2018) 619-626.

[10] S. Morin, E. Rahme, H. Behlouli, A. Tenenhouse, D. Goltzman, L. Pilote, Effectiveness of antiresorptive agents in the prevention of recurrent hip fractures, Osteoporos Int 18(12) (2007) 1625-32.

[11] J.A. Kanis, A. Oden, O. Johnell, H. Johansson, C. De Laet, J. Brown, P. Burckhardt, C. Cooper, C. Christiansen, S. Cummings, J.A. Eisman, S. Fujiwara, C. Glüer, D. Goltzman, D. Hans, M.A. Krieg, A. La Croix, E. McCloskey, D. Mellstrom, L.J. Melton, 3rd, H. Pols, J. Reeve, K. Sanders, A.M. Schott, A. Silman, D. Torgerson, T. van Staa, N.B. Watts, N. Yoshimura, The use of clinical risk factors enhances the performance of BMD in the prediction of hip and osteoporotic fractures in men and women, Osteoporos Int 18(8) (2007) 1033-46.

[12] J. Sheng, X. Qu, X. Zhang, Z. Zhai, H. Li, X. Liu, H. Li, G. Liu, Z. Zhu, Y. Hao, A. Qin, K. Dai, Coffee, tea, and the risk of hip fracture: a meta-analysis, Osteoporos Int 25(1) (2014) 141-50.

[13] P. Vestergaard, L. Mosekilde, Hyperthyroidism, bone mineral, and fracture risk--a meta-analysis, Thyroid 13(6) (2003) 585-93.

[14] J. Hippisley-Cox, C. Coupland, Predicting risk of osteoporotic fracture in men and women in England and Wales: prospective derivation and validation of QFractureScores, Bmj 339 (2009) b4229.

[15] N. Goto, A. Weststrate, F. Oosterlaan, M. Verhaar, H. Willems, M. Emmelot-Vonk, M. Hamaker, The association between chronic kidney disease, falls, and fractures: a systematic review and meta-analysis, Osteoporosis international 31 (2020) 13-29.

[16] J.A. Kanis, Diagnosis of osteoporosis and assessment of fracture risk, Lancet 359(9321) (2002) 1929-36.

[17] T.N. Poly, M.M. Islam, H.C. Yang, C.C. Wu, Y.J. Li, Proton pump inhibitors and risk of hip fracture: a meta-analysis of observational studies, Osteoporos Int 30(1) (2019) 103-114.

[18] N. Wang, Y. Chen, J. Ji, J. Chang, S. Yu, B. Yu, The relationship between serum vitamin D and fracture risk in the elderly: a meta-analysis, J Orthop Surg Res 15(1) (2020) 81.

[19] L. Vandenput, K. Sjögren, J. Svensson, C. Ohlsson, The role of IGF-1 for fracture risk in men, Front Endocrinol (Lausanne) 3 (2012) 51.

[20] T. Tanaka, H. Matsumoto, B.K. Son, S. Imaeda, E. Uchiyama, S. Taniguchi, A. Nishino, T. Miura, T. Tanaka, T. Otsuki, K. Nishide, K. Iijima, J. Okata, Environmental and physical factors predisposing middle-aged and older Japanese adults to falls and fall-related fractures in the home, Geriatr Gerontol Int 18(9) (2018) 1372-1377.

[21] C. Temml, A. Ponholzer, G. Gutjahr, I. Berger, M. Marszalek, S. Madersbacher, Nocturia is an age-independent risk factor for hip-fractures in men, Neurourol Urodyn 28(8) (2009) 949-52.

[22] K. Donnelly, R. Bracchi, J. Hewitt, P.A. Routledge, B. Carter, Benzodiazepines, Z-drugs and the risk of hip fracture: A systematic review and meta-analysis, PLoS One 12(4) (2017) e0174730.

[23] H.J. Kim, H.J. Chun, C.D. Han, S.H. Moon, K.T. Kang, H.S. Kim, J.O. Park, E.S. Moon, B.R. Kim, J.S. Sohn, S.Y. Shin, J.W. Jang, K.I. Lee, H.M. Lee, The risk assessment of a fall in patients with lumbar spinal stenosis, Spine (Phila Pa 1976) 36(9) (2011) E588-92.

[24] T. Suzuki, H. Yoshida, T. Hashimoto, N. Yoshimura, S. Fujiwara, M. Fukunaga, T. Nakamura, K. Yoh, T. Inoue, T. Hosoi, H. Orimo, Case-control study of risk factors for hip fractures in the Japanese elderly by a Mediterranean Osteoporosis Study (MEDOS) questionnaire, Bone 21(5) (1997) 461-7.

[25] H. Iida, T. Seki, Y. Takegami, Y. Osawa, D. Kato, G. Takemoto, K. Ando, S. Ishizuka, Y. Hasegawa, S. Imagama, Association between locomotive syndrome and fall risk in the elderly individuals in Japan: The Yakumo study, J Orthop Sci 29(1) (2024) 327-333.

[26] A. Ferrer, F. Formiga, O. Plana-Ripoll, M.A. Tobella, A. Gil, R. Pujol, Risk of falls in 85-year-olds is associated with functional and cognitive status: the Octabaix Study, Arch Gerontol Geriatr 54(2) (2012) 352-6.

[27] N.C. Harvey, A. Odén, E. Orwoll, J. Lapidus, T. Kwok, M.K. Karlsson, B.E. Rosengren, E. Ribom, C. Cooper, P.M. Cawthon, J.A. Kanis, C. Ohlsson, D. Mellström, H. Johansson, E. McCloskey, Measures of Physical Performance and Muscle Strength as Predictors of Fracture Risk Independent of FRAX, Falls, and aBMD: A Meta-Analysis of the Osteoporotic Fractures in Men (MrOS) Study, J Bone Miner Res 33(12) (2018) 2150-2157.

[28] M. Uragami, K. Matsushita, Y. Shibata, S. Takata, T. Karasugi, T. Sueyoshi, T. Masuda, T. Nakamura, T. Tokunaga, S. Hisanaga, M. Yugami, K. Sugimoto, R. Yonemitsu, K. Ideo, Y. Fukuma, K. Takata, T. Arima, J. Kawakami, K. Maeda, N. Yoshimura, H. Matsunaga, Y. Kai, S. Tanimura, M. Shimada, M. Tateyama, K. Miyamoto, R. Kubo, R. Tajiri, X. Tian, F. Homma, J. Morinaga, Y. Yamanouchi, M. Takebayashi, N. Kajitani, Y. Uehara, G. Kumamoto Stop OsteoPorotic Hip Fractures, T. Miyamoto, A machine learning-based scoring system and ten factors associated with hip fracture occurrence in the elderly, Bone 176 (2023) 116865.

Supplementary table 2. In univariate Cox proportional hazards regression analysis, for factors that were significant, the p-value for each factor was adjusted using the Benjamini–Hochberg method for false discovery rate (FDR) correction, and the corresponding q-value was calculated.

| Variables | p-value | q-value |
| --- | --- | --- |
| living alone | 0.0085 | 0.0340 |
| three or more exsting vartebral fracture | 0.0220 | 0.0440 |
| Hypertension | 0.0408 | 0.0462 |
| low maximal grip strength(<18kg) | 0.0462 | 0.0462 |

Supplementary table 3. Basic Patient Characteristics.

| Characteristics | Controls  (N=888) | Cases  (N=31) | P-value |
| --- | --- | --- | --- |
| Sex, number (%) |  |  | 0.030 |
|  |  |  |  |
| Male | 165 (18.6) | 1(3.2) |  |
| Female | 723(81.4) | 30(96.8) |  |
| Age, years (mean (SD)) | 83.6 (9.3) | 86.4 (7.2) | 0.047 |
| BMI (kg/m²) (mean (SD)) | 20.9 (3.5) | 20.3 (3.4) | 0.381 |
| Facture pattern, number (%) |  |  | 0.017 |
| Femoral neck fracture | 431(48.5) | 22(71.0) |  |
| Femoral intertrochanteric fracture | 457(51.5) | 9(29.0) |  |
| Living in a nursing home, number (%) | 220(24.8) | 6(19.4) | 0.671 |
| Living alone, number (%) | 154(17.4) | 13(41.9) | 0.002 |
| Walking with an aid, number (%) | 405(45.8) | 17(54.8) | 0.362 |
| With Long-term Care Insurance (LTCI) certification, number (%) | 544(61.3) | 23(74.2) | 0.188 |
| Number of falls in previous 12 months, number (%) |  |  | 0.113 |
| None | 339(43.8) | 8(30.8) |  |
| 1 | 156(20.2) | 8(30.8) |  |
| 2 | 114(14.7) | 7(26.9) |  |
| 3 or more | 165(21.3) | 3(11.5) |  |
| Hours per week spent walking, number (%) |  |  | 0.385 |
| unable to walk | 37(4.7) | 0(0) |  |
| <1 | 205(26.2) | 9(36) |  |
| 1-4 | 165(21.1) | 7(28) |  |
| ≥4 | 374(47.9) | 9(36) |  |
| Levels of cognitive impairment |  |  | 0.813 |
| No significant | 283(32.2) | 7(22.6) |  |
| mild cognitive impairment | 289(32.9) | 12(38.7) |  |
| somewhat difficult to communicate | 210(23.9) | 9(29.0) |  |
| difficulty in communicating | 59(6.7) | 2(6.5) |  |
| communication extremely difficult | 30(3.4) | 1(3.2) |  |
| completely unable to communicate | 7(0.8) | 0(0) |  |
| Barthel index score (mean (SD)) | 72.2(26.0) | 76.7(20.1) | 0.254 |
| GLSF-25 score (mean (SD)) | 43.6(25.2) | 43.8(19.7) | 0.972 |
| LS stage 3, number (%) | 622(73.3) | 26(83.9) | 0.219 |
| LS stage 2 or higher, number (%) | 694(81.8) | 29(93.5) | 0.146 |
| LS stage 1 or higher, number (%) | 782(92.1) | 31(100) | 0.161 |
| Maximal handgrip strength |  |  | 0.193 |
| <18kg | 558(74.8) | 25(86.2) |  |
| 18kg or more | 118(25.2) | 4(13.8) |  |
| Glucocorticoid use, number (%) | 25(2.8) | 1(3.2) | 0.595 |
| Alcohol intake >3 units daily, number (%) | 25(2.8) | 0(0) | 1.000 |
| Smoking status, number (%) |  |  | 0.936 |
| non-smoker | 728(82.3) | 27(87.1) |  |
| current smoker | 27(3.1) | 1(3.2) |  |
| smoking cessation < 10 years | 39(4.4) | 1(3.2) |  |
| smoking cessation ≥ 10 years | 91(10.3) | 2(6.5) |  |
| Cups of tea/day, number (%) |  |  | 0.879 |
| None | 43(4.9) | 1(3.2) |  |
| One | 51(5.8) | 2(6.5) |  |
| Two | 101(11.6) | 3(9.7) |  |
| Three | 267(30.5) | 10(32.3) |  |
| Four | 131(15.0) | 7(22.6) |  |
| more than five | 281(32.2) | 8(25.8) |  |
| Type of bed, number (%) |  |  | 0.341 |
| Western bed | 717(81.5) | 28(90.3) |  |
| Japanese futon | 163(18.5) | 3(9.7) |  |
| Nocturia, number (%) |  |  | 0.752 |
| no waking up during the night to urinate | 72(8.2) | 2(6.5) |  |
| waking up once or twice | 466(52.8) | 18(58.1) |  |
| waking up three or four times | 216(24.5) | 6(19.4) |  |
| waking up more than five times | 47(5.3) | 3(9.7) |  |
| use of a diaper | 81(9.2) | 2(6.5) |  |
| Previous fragility fractures, number (%) | 10(52.7) | 27(67.7) | 0.142 |
| Number of existing vertebral compression fractures, number (%) |  |  |  |
| one or more | 430(48.7) | 19(61.3) | 0.202 |
| Two or more | 233(26.4) | 12(38.7) | 0.148 |
| Three or more | 105(11.9) | 7(22.6) | 0.090 |
| Femoral neck T-score (mean (SD)) | -3.14(1.24) | -3.43(0.99) | 0.146 |
| eGFR (ml/min/1.73 ㎡) (mean (SD)) | 66.4(23.6) | 57.1(17.4) | 0.006 |
| Serum 25(OH)D (ng/ml) (mean (SD)) | 11.14(8.16) | 9.32(4.36) | 0.039 |
| Serum IGF-1 (ng/ml) (mean (SD)) | 56.5(28.2) | 48.8(17.5) | 0.024 |
| Comorbidity, number (%) |  |  |  |
| Cerebrovascular disease | 180(20.4) | 4(12.9) | 0.370 |
| Parkinson's disease | 26(2.9) | 0(0) | 1.000 |
| Visual impairment | 165(18.1) | 5(16.1) | 1.000 |
| Cardiovascular disease | 192(21.7) | 5(16.1) | 0.656 |
| Respiratory disease | 135(15.3) | 3(9.7) | 0.608 |
| Chronic liver disease | 32(3.6) | 0(0) | 0.622 |
| Chronic kidney disease | 46(5.2) | 1(3.2) | 1.000 |
| Hypertension | 593(67) | 26(83.9) | 0.052 |
| Hyperthyroidism | 30(3.4) | 1(3.2) | 1.000 |
| Dyslipidemia |  |  |  |
| Serum LDL-cholesterol≧140mg/dL | 41(4.7) | 1(3.2) | 1.000 |
| Serum triglyceride≧150mg/dL | 152(17.5) | 6(19.4) | 0.810 |
| Serum HDL-cholesterol＜40mg/dL | 484(55.6) | 12(38.7) | 0.069 |
| Diabetes | 121(13.7) | 3(9.7) | 0.788 |
| Lumbar spinal canal stenosis | 71(8) | 5(16.1) | 0.172 |
| Rheumatoid arthritis | 33(3.7) | 1(3.2) | 1.000 |
| Secondary osteoporosis | 128(14.6) | 7(22.6) | 0.207 |
| Gastric surgery | 39(4.4) | 0(0) | 0.640 |
| Parental history of hip fracture, number (%) | 67(11.4) | 0(0) | 0.151 |
| Use of hypnotics, number (%) | 258(29.2) | 9(29) | 1.000 |
| Proton pump inhibitor use, number (%) | 289(32.5) | 11(35.5) | 0.702 |
| Use of anti-osteoporosis drugs, number (%) |  |  |  |
| Before initial fracture | 192(21.6) | 8 (25.8) | 0.657 |
| At time of discharge from hospital | 467(52.6) | 17(54.8) | 0.856 |

Supplementary table 4. Logistic regression analysis adjusted by age, sex, and BMI.

| Variables | OR | 95%CI | p-value |
| --- | --- | --- | --- |
| Fracture pattern (Femoral neck fracture) | 3.29 | 1.460-7.430 | 0.0042 |
| Living alone | 3.60 | 1.710-7.580 | 0.0007 |
| eGFR | 0.982 | 0.965-0.999 | 0.0336 |

Only significant factors are listed.

Supplementary Table 5. Multivariate logistic regression analysis with factors significant in two-group comparisons or in logistic regression analysis as explanatory variables.

| Variables | OR | 95%CI | p-value |
| --- | --- | --- | --- |
| Age | 1.05 | 0.991-1.120 | 0.098 |
| Sex (female) | 5.24 | 0.685-40.000 | 0.111 |
| Facture pattern (Femoral neck fracture) | 4.36 | 1.830-10.400 | 8.65e-4 |
| Living alone | 3.99 | 1.820-8.760 | 5.43e-4 |
| eGFR | 0.976 | 0.957-0.995 | 0.012 |
| Serum 25(OH)D | 0.964 | 0.892-1.040 | 0.357 |
| Serum IGF-1 | 0.988 | 0.971-1.000 | 0.164 |
