# Supplementary material for: Identification of factors predictive of contralateral secondary hip fractures in patients after initial hip fracture - A prospective observational study
Source: Bone Rep. 2026 Feb 19;29:101908. doi: 10.1016/j.bonr.2026.101908 (PMC12973514; doi:10.1016/j.bonr.2026.101908)
Supplement: Supplementary file 2 — Fig. S1 Patient flowchart. We enrolled 1395 hip fracture patients and followed them for 1 year. Excluded were those with a history of contralateral hip fracture, those who died within 1 year of the initial fracture, and those who could not be followed for various other indicated reasons. Of the remaining 919 patients, 31 sustained contralateral hip fractures in the follow-up year, while 888 did not. Fig. S2 Cut-off value for eGFR levels significantly associated with occurrence of secondary hip fracture within 1 year. Cut-off values for eGFR levels significantly associated with occurrence of secondary hip fracture within 1 year were 59 ml/min/1.73m2, based on the Receiver Operating Characteristic (ROC) curve. Area Under Curve (AUC) was determined to be 0.628 (95% CI, 0.539–0.716). [file mmc2.pptx]

## Slide 1
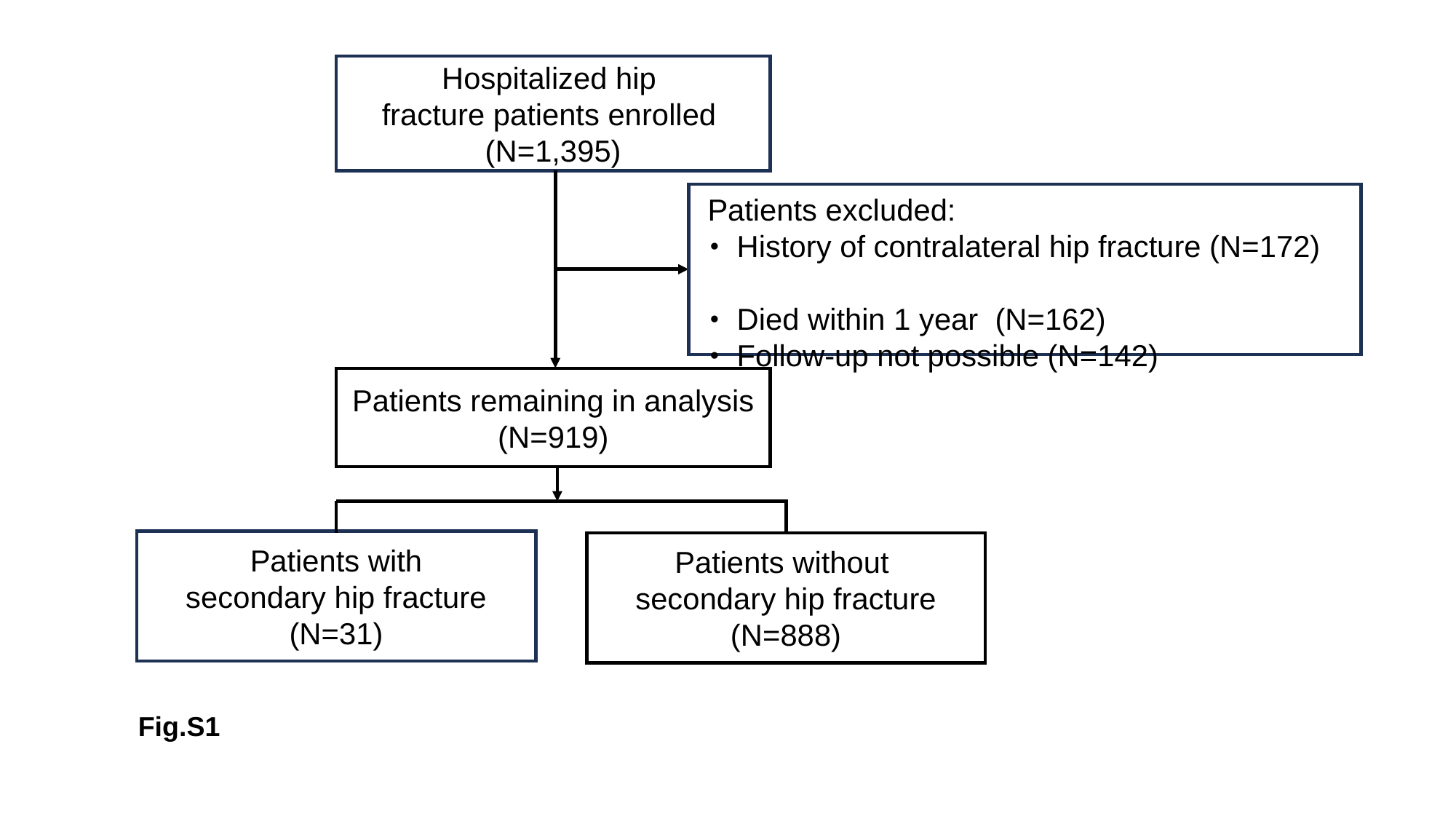

Hospitalized hip
fracture patients enrolled
(N=1,395)
Patients remaining in analysis
(N=919)
Patients with
secondary hip fracture
(N=31)
Patients without
secondary hip fracture
(N=888)
 Patients excluded:
・History of contralateral hip fracture (N=172)
・Died within 1 year (N=162)
・Follow-up not possible (N=142)
Fig.S1

## Slide 2
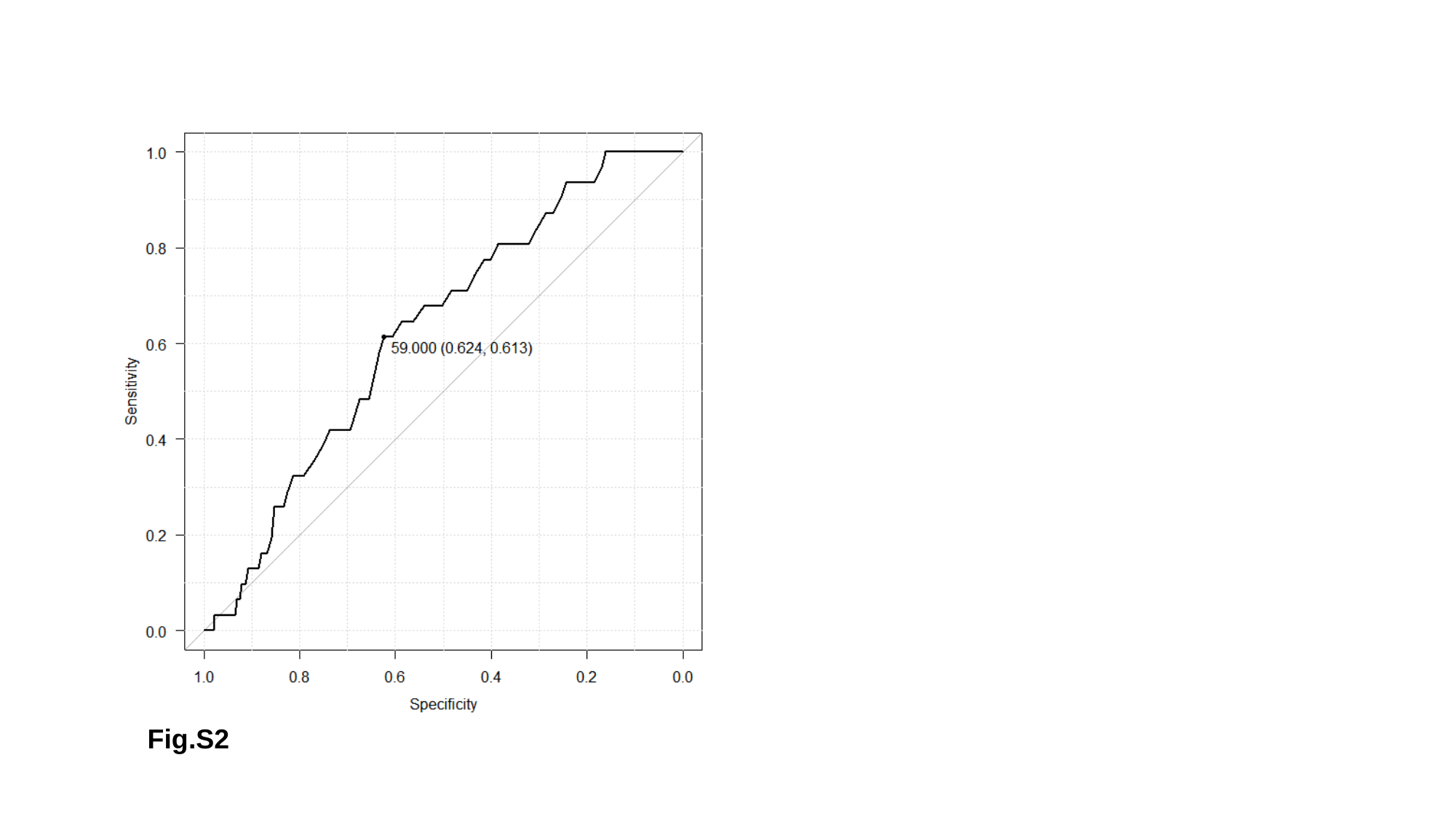

Fig.S2
